# Survival Outcomes and Genetic Characteristics of Resected Pancreatic Acinar Cell Carcinoma

**DOI:** 10.1245/s10434-024-16331-4

**Published:** 2024-11-22

**Authors:** Alex B. Blair, Shannon N. Radomski, Joanne Chou, Mengyuan Liu, Thomas Clark Howell, Wungki Park, Eileen M. O’Reilly, Lei Zheng, Vinod P. Balachandran, Alice C. Wei, T. Peter Kingham, Michael I. D’Angelica, Jeffrey Drebin, Sabino Zani, Dan G. Blazer, Richard A. Burkhart, William R. Burns, Kelly J. Lafaro, Peter J. Allen, William R. Jarnagin, Michael E. Lidsky, Jin He, Kevin C. Soares

**Affiliations:** 1 Present Address: Department of Surgery, Memorial Sloan Kettering Cancer Center, New York, NY USA; 2 Department of Surgery, Johns Hopkins Hospital, Johns Hopkins University School of Medicine, Baltimore, MD USA; 3 Department of Biostatistics, Memorial Sloan Kettering Cancer Center, New York, NY USA; 4 Department of Surgery, Duke University Hospital, Durham, NC USA; 5 Department of Gastrointestinal Oncology, Memorial Sloan Kettering Cancer Center, New York, NY USA; 6 Department of Oncology, Johns Hopkins Hospital, Baltimore, MD USA; 7 Department of Surgery, The Ohio State University Wexner Medical Center, Columbus, OH USA

**Keywords:** Acinar cell carcinoma, Pancreatic exocrine cancer, Homologous repair deficiency, Whole exome sequencing, Survival

## Abstract

**Background:**

Pancreatic acinar cell carcinoma (pACC) is a rare neoplasm of the exocrine pancreas. There is a dearth of information about tumor characteristics and patient outcomes. This study describes the clinical characteristics, genetic alterations, and survival outcomes of resected pACC.

**Patients and Methods:**

Consecutive patients undergoing pancreatectomy for pathologically confirmed pACC from 1999 to 2022 across three high-volume pancreas surgery centers were analyzed. Patient demographics, tumor characteristics, treatment data, and genetic sequencing were obtained through retrospective abstraction.

**Results:**

A total of 61 patients with resected pACC were identified. Median overall survival (OS) was 73 months and median recurrence free survival was 22 months. Nine patients underwent resection for oligometastatic disease; median OS was not reached after a median follow-up of 31 months from date of metastasectomy. Adjuvant chemotherapy was administered in 67% of patients with FOLFOX/FOLFIRINOX (5-fluorouracil, leucovorin, oxaliplatin, ± irinotecan) the most common regimen (58%). Sequencing data were obtained in 47 (77%) patients. A mutation in at least one of three core genes associated with the homologous recombination repair (HRR) pathway (*BRCA1*, *BRCA2*, or *PALB2*) occurred in 26% (*n* = 12) with *BRCA2* the most frequently identified. A mutation in any other “non-core” gene associated with DNA damage repair or the HRR pathway was identified in 45% (*n* = 21) with a high tumor mutational burden of > 10 mutations per megabase in 13%.

**Conclusions:**

Resection of pACC is associated with favorable survival outcomes, even in the setting of oligometastatic disease. Mutations in the HRR pathway are common, providing opportunities for potential targeted therapeutic options.

**Supplementary Information:**

The online version contains supplementary material available at 10.1245/s10434-024-16331-4.

Pancreatic acinar cell carcinoma (pACC) is a rare malignant subtype representing only 1–2% of pancreatic tumors.^1–3^ Despite acinar tissue making up most of the exocrine pancreas, adenocarcinomas of the ductal cells (PDAC) represent the overwhelming majority of tumors.^4^ Due to the rarity of pACC, there is a paucity of data on clinical features, tumor characteristics, and survival outcomes; thus, there is a lack of knowledge to inform optimal therapy for these patients. Prior case series have suggested survival outcomes are more favorable in pACC than standard PDAC, with complete surgical resection associated with the highest rates of long-term survival.^5–8^ However, beyond recommended surgical extirpation when feasible, treatment strategies are largely based on the well-established PDAC experience. Thus, there are currently no clear guidelines for optimal chemotherapeutic regimens for patients with pACC.

The genetic heterogeneity of PDAC and low prevalence of targetable mutations is thought to play a role in its relative therapeutic resistance.^9^ Nevertheless, a cluster of mutations involved in double-stranded DNA homologous recombination repair (HRR) have been identified in 3–7% of patients with PDAC.^10–13^ Core genetic mutations associated with HRR include *BRCA1*, *BRCA2*, and *PALB2* and are of increasing relevance in treatment selection.^14,15^ DNA maintenance gene inactivation and subsequent repair deficiency in patients with these mutations may impart sensitivity to DNA strand damaging cytotoxic agents such as poly ADP-ribose polymerase inhibitor (PARPi) or platinum-based chemotherapy.^16,17^ The use of platinum chemotherapy and PARPi has conveyed a survival advantage in PDAC patients with core HRR mutations but the impact on outcomes in pACC remain unclear.^18–21^ In a large pan-cancer cohort whom underwent matched tumor/normal sequencing, patients with pACC of any stage harbored germline pathogenic variants in HRR at greater rates than PDAC alternatives, suggesting pACC should be considered as part of the spectrum of *BRCA-*related malignancies.^22^ These data further support a need for further investigation into the genetic characteristics of pACC and incidence of HRR mutations within the resected cohort.

The main objectives of this multi-institutional retrospective study are to describe the genetic characteristics and characterize the cancer-related outcomes of patients with resected pACC. Additionally, the incidence of HRR mutations within the present cohort of patients with resected pACC is investigated.

## PATIENTS AND METHODS

### Patient Cohort

Clinicopathologic data for patients undergoing primary pancreatic resection at Johns Hopkins Hospital (JHH), Memorial Sloan Kettering Cancer Center (MSK), and Duke University Hospital between 1999 and 2022 were collected and included in this study. Inclusion criteria were a diagnosis of pACC upon final histopathologic analysis confirmed by institutional pathologists. Outcomes of clinical interest include overall survival (OS), recurrence free survival (RFS), and survival after metastasesectomy. Follow-up data were retrieved from institutional databases. The date of death was obtained from medical records, local obituary or the Social Security Death Index. OS was calculated from the date of initial surgery to the date of death from any cause or censored at the date of last follow-up. RFS was calculated from the date of surgery to the date of suspected recurrence on cross-sectional imaging, death from any cause, or censored at the date of last follow-up. The first location of disease recurrence was documented based on surveillance imaging; histologic confirmation with biopsy was not required. Institutional review board approval was obtained for this retrospective study.

### Demographics and Clinicopathological Characteristics

All patient data were collected from independent institutional databases and shared via a deidentified protected dataset per institutional guidelines. Patient demographics included age, sex, and preoperative carbohydrate antigen (CA)19-9. Serum lipase levels were not available in the majority of patients and thus were not included in analyses. Neoadjuvant and adjuvant chemotherapy and/or radiation were noted. The type of surgery was selected based on the location of the primary tumor and discretion of treatment surgeon and included pancreaticoduodenectomy, distal pancreatectomy, or total pancreatectomy. Tumor pathology characteristics including tumor size, stage, perineural invasion, lymphovascular invasion, presence of nodal disease, and margin status were extracted from final pathology reports. Resections were considered “R0” when malignant cells were greater than 1 mm from the surgical margin. Resection of oligometastatic disease was clarified as at the time of primary pancreas resection, or after time of primary pancreas resection. Surgical resection of metastases was carefully considered in a multidisciplinary setting on a case-by-case basis.

Treatment data were abstracted from medical records. All radiation therapy in this study when utilized was conventional external beam radiotherapy (EBRT). Chemotherapy regimens were selected at the discretion of the treating medical oncologist and were broadly defined as gemcitabine-based regimen (predominately gemcitabine and nab-paclitaxel or gemcitabine and capecitabine), FOLFOX/FOLFIRINOX (5-fluorouracil, leucovorin, oxaliplatin, ± irinotecan), capecitabine alone, or the sequential combination of both gemcitabine-based therapy and platinum-based therapies (FOLFIRINOX or cisplatin).

### Genetic Data

Tissue from the resected pancreatic tumor specimen was utilized for collection of genomic data. Memorial Sloan Kettering Cancer Center Integrated Mutation Profiling of Actionable Cancer Targets (MSK-IMPACT) is an institutional protocol yielding genomic data and profiling that was leveraged for a majority of patients in this study.^23^ Whole exome sequencing was performed on a previously published cohort included in this study.^24^ Remaining patients had commercial or institutional sequencing per treating medical oncologist’s recommendations, with results abstracted from medical records. Core HRR mutations were considered to be *BRCA1*, *BRCA2*, or *PALB2*.^14^ Noncore HRR mutations include: *ATM*, *BAP1*, *BARD1*, *BLM*, *BRIP1*, *CHEK2*, *FAM175A*, *FANCA*, *FANCC*, *NBN*, *RAD50*, *RAD51*, *RAD51C*, and *RTEL1.*^25,26^ Patients with pathogenic or likely pathogenic variants were included, while patients with variants of unknown significance were excluded.^27^

### Statistical Analysis

Statistical analyses were conducted using R version 4.2.2 with the tidyverse (v2.0.0) and gtsummary (v1.7.1) packages (@r, @tidyverse, @gtsummary). Categorical variables were expressed as percentages. Continuous variables were presented as median and interquartile range (IQR). Survival analysis was performed using Kaplan–Meier survival estimates.

## RESULTS

A total of 61 patients underwent pancreatic resection for pACC at JHH (*n* = 32), MSK (*n* = 22), and Duke University Hospital (*n* = 7) from 1999 to 2022 and were included in this study. Median follow up for the cohort was 34 months (IQR: 10–60). Demographic and clinicopathologic characteristics for all included patients are summarized in Table 1. The majority (70%) were male with a median age of 63 years (IQR: 56–71) at the time of operation. A similar number of patients underwent pancreaticoduodenectomy (52%) and distal pancreatectomy (44%) with only two undergoing a total pancreatectomy (3%). The median primary tumor size was 4.5 cm (IQR: 2.8–7.3) based on the final pathology report of the resected specimen. Nodal metastasis was identified in 39% of patients. Evidence of perineural and lymphovascular invasion were observed in 53% and 56% respectively. The overall R0 resection rate was 87%.

**TABLE 1:** Clinicopathologic characteristics

Variable	*n* = 61	
Age, median years [IQR]	63	[55, 71]
Male, *n* (%)	43	70%
CA19-9, median [IQR]	22	[8, 45]
Operation procedure, *n* (%)		
Pancreaticoduodenectomy	32	52%
Distal pancreatectomy	27	44%
Total pancreatectomy	2	3%
Oligometastasectomy		
At time of initial surgery	6	10%
Following initial surgery	3	5%
Size, median cm [IQR]	4.7	[2.8, 7.5]
T stage		
1	5	8%
2	23	38%
3	33	54%
Nodal stage		
0	37	61%
1	24	39%
Perineural invasion	32	53%
Lymphovascular invasion	34	56%
R0 resection	53	87%
Treatment site		
Johns Hopkins Hospital	32	52%
Memorial Sloan Kettering Cancer Center	22	36%
Duke University Hospital	7	12%

### Treatment Characteristics

Chemotherapy and radiation regimens administered in the treatment of resected pACC patients in this cohort are summarized in Table 2. A quarter of patients received neoadjuvant chemotherapy, of which FOLFIRINOX was the most frequently utilized regimen (67%). Neoadjuvant EBRT was utilized in only 5% of patients. Adjuvant chemotherapy was delivered to 67% of patients after resection. Adjuvant chemotherapy treatment regimens included FOLFIRINOX (15/41) or FOLFOX (2/41), gemcitabine-based regimens (11/41), capecitabine alone (2/41), or multiple therapies (11/41), which typically involved sequential combinations of gemcitabine and FOLFIRINOX or gemcitabine, cisplatin ± capecitabine. Adjuvant radiation was administered to seven patients (13%).

**TABLE 2:** Treatment characteristics

Variable	*n = 61*	
Neoadjuvant chemotherapy	15	25%
Neoadjuvant chemotherapy regimen		
Gemcitabine	2	13%
FOLFIRINOX	10	67%
Capecitabine	2	13%
Unknown	1	7%
Neoadjuvant radiation	3	5%
Adjuvant chemotherapy		
Yes	41	67%
No	13	21%
Unknown	7	–
Adjuvant chemotherapy reg		
None	13	21%
Gemcitabine*	11	18%
FOLFOX/FOLFIRINOX#	17	28%
Capecitabine	2	3%
Sequential combination@	11	18%
Unknown	7	–
Adjuvant radiation	7	13%

^*^Gemcitabine based combinations include Gem Abraxane (3), Gem Xeloda (6), Single agent Gem (2)

^#^FOLFOX (2) FOLFIRINOX (15)

@Gemcitabine + Cisplatin ± Xeloda (6), Gemcitabine + FOLFIRINOX/FOLFOX (5)

### Genetic Sequencing

Genetic sequencing data were available in 47/61 patients (77%). Mutation data are summarized in Table 3. A total of 14 core HRR mutations were identified in 12 resected specimens (26%). These included *BRCA2* (*n* = 10), *BRCA1* (*n* = 2), and *PALB2* (*n* = 2). Noncore HRR mutations were identified in 11 tumors, including *ATM* (*n* = 4), *FANCA* (*n* = 2), *RAD50* (*n* = 2), and 1 each of *FAM175A, FANCC,* and *BLM*. Co-occurring alterations were identified in two patients with one patient having both *BRCA2* and *ATM* mutations and the other having both *BRCA2* and *FAM175A*. Tumor mutational burden was available in 38 resected specimens with a median of 2.6 mutations per megabase (IQR: 1.0–4.4) and range from 0.5 to 22.6. A tumor mutational burden >10 was observed in 5 specimens (13%).

**Table 3:** Mutation data of resected pACC specimen that underwent sequencing

Variable	*n* = 47	
Core HRR mutation: *BRCA1, BRCA2, PALB2*	12	26%
Non-Core HRR mutation: *ATM, BAP1, BARD1, BLM, BRIP1, CHEK2, FAM175A, FANCA, FANCC, NBN, RAD50, RAD51, RAD51C, RTEL1*	11	23%
Any HRR mutation:	21	45%
Tumor mutation burden, median [IQR]	2.6	[1.0, 4.4]
Tumor mutation burden > 10	5	13%

Core *HRR* mutations	*n* =12	
BRCA1*	2	17%
BRCA2*	10	83%
*PALB2*	2	17%

Non-core* HRR* mutations	*n* =11	
*ATM*	4	36%
*FAM175A*	1	9%
*FANCA*	2	18%
*FANCC*	1	9%
*BLM*	1	9%
*RAD50*	2	18%

^*^n = 1 with both *BRCA1* and *BRCA2* mutation

^**^n = 1 with both *BRCA1* and *PALB2* mutation

### Recurrence and Survival

**FIG. 1:**
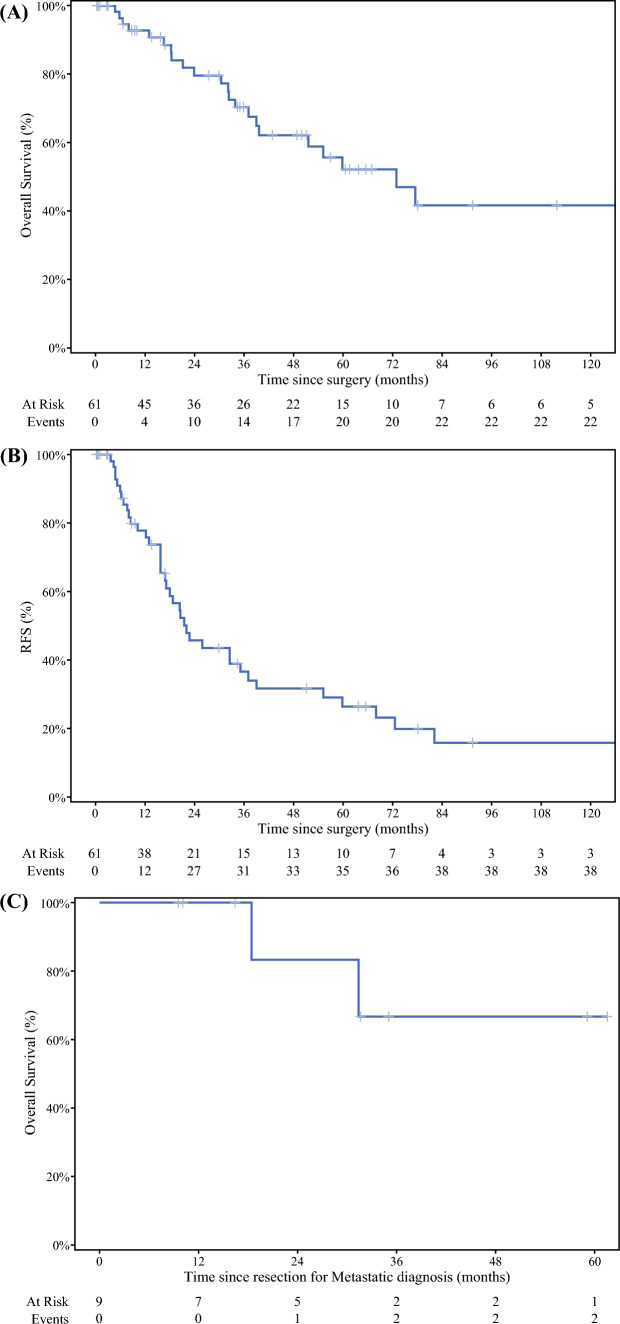
Kaplan–Meier survival estimates of resected pACC disease from the time of surgery: **A** overall survival, **B** recurrence free survival, **C** overall survival from time of oligometastatic disease resection

Survival curves are shown in Fig. 1. At a median follow-up of 35 months (range: 1–224), the median OS for the cohort was 73 months from surgical resection (Fig. 1A). Disease recurrence or death was documented in 39 of 61 patients (64%) with a median RFS of 22 months from surgical resection (Fig. 1B). The 5-year OS for the cohort was 52% with a 5-year RFS of 20%. A subset of nine patients underwent resection for oligometastatic disease. Of these, six were resected at the time of the primary and three patients underwent metastasectomy of stable disease after additional adjuvant treatment. Sites of metastatic disease resection included liver (*n* = 4), peritoneum (*n* = 3), remnant pancreas (*n* = 1), and soft tissue/buttock (*n* = 1). Median OS as calculated from the date of metastasectomy to death or last follow-up was not reached, with a median follow up of 31 months (IQR: 16–35) (Fig. 1C).

**FIG. 2:**
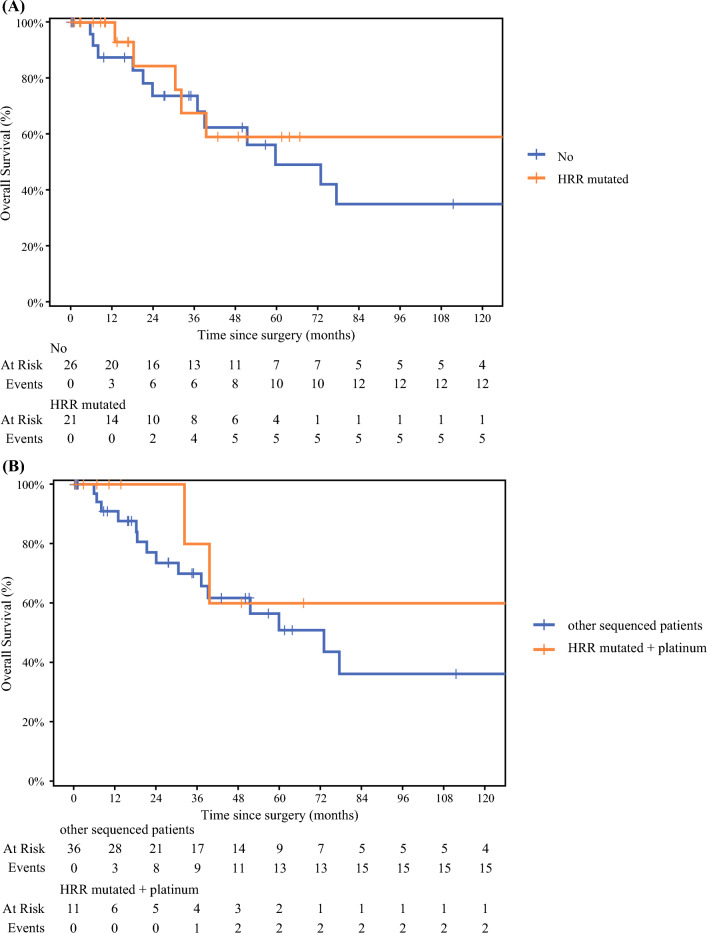
Kaplan–Meier survival estimates of resected pACC disease comparing **A** patients with pACC with a deleterious core and noncore mutation in the HRR pathway versus pACC patients with no mutation **B** with a deleterious mutation in the core HRR pathway whom received adjuvant platinum therapy versus all other sequenced patients

The presence of any mutation involving the core HRR or noncore pathways was not statistically associated with survival outcomes (Fig. 2A). Median overall survival was not met for patients with a core HRR gene mutation who received platinum-based therapy at any time, however, this did not reach significance compared to the remaining cohort of sequenced patients (median OS not met versus 73 months, *p* = 0.512) (Fig. 2B).

**FIG. 3:**
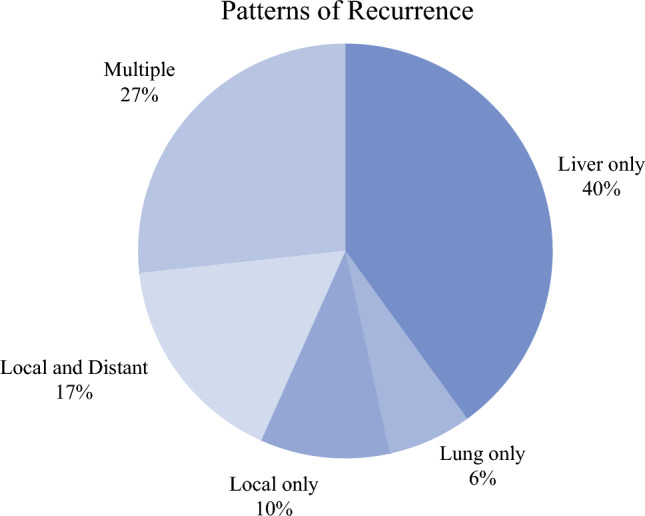
Distribution of documented recurrence patterns after resection of pACC

The first site of disease recurrence on post-resection surveillance imaging was documented and divided into five categories: (1) local recurrence or disease in the remnant pancreas or surgical bed, (2) liver-only disease, (3) lung-only disease, (4) simultaneous local and distant disease, or (5) multiple sites of distant recurrence, including carcinomatosis. A distribution of these patterns of recurrence is detailed in Fig. 3. Of patients that recurred, isolated hepatic disease as the initial site of documented recurrence was the most frequently observed pattern (40%) while isolated pulmonary metastases were uncommon (6%). Local only or simultaneous local and distant disease recurrence were observed in 10% and 17% of patients, respectively. These patterns of recurrence were similar within the cohort of pACC with core HRR mutations; isolated hepatic disease was the most frequently observed (33%) while local only (17%) and isolated pulmonary metastases (0%) were the least common (Supplementary Fig. 1).

## DISCUSSION

In the current study, we report on the largest series of resected pACC with descriptive sequencing data from three high-volume pancreatic surgery institutions. Patients with pACC who were able to undergo resection had favorable survival outcomes with a median OS of > 70 months. Mutations associated with the HRR pathway were identified in 45% of specimen and a core HRR mutation in 26%. These data suggest opportunities for potentially informed targeted therapeutic options for patients with pACC. Routine genetic sequencing in this patient population may assist not only with patient therapy, but also identification of family with HRR gene alterations for screening and early detection of multiple malignancies.

Often pACC are large in size relative to that of PDAC at the time of diagnosis with many described as > 10cm and a median size of approximately 5 cm on cross sectional imaging.^28,29^ Consistent with this, the median tumor size across the present study was 4.7 cm with seven (11%) patients having a tumor > 10 cm at the time of resection.

In this study we report a median OS of 73 months from the time of surgery and median RFS of 22 months. This is similar to other institutional series and survival estimates for pACC amenable to surgery, and far superior to the unresectable, metastatic cohort not discussed in this study but reported to be a median of 15 months in other institutional experiences.^30^ Matos et al. described 15 patients with resected pACC with a median OS of 61 months.^6^ A recent multi-institutional series of 66 cases of pACC across the Massachusetts General Brigham Healthcare system described a median OS of 24 months across the whole cohort, but enhanced OS to 42 months in the 36 patients with localized disease treated with surgery.^7^ There are currently limited reports of RFS estimates following resection of pACC. A multi-institutional European series of 59 patients reported a median RFS of 30 months for resected patients of which 24/31 had distant recurrence.^8^ Despite encouraging OS, median RFS for the present reported cohort was 22 months. We found that 64% (39/61) of patients with resected pACC had documented recurrence or death at a median follow up of 35 months from surgery. The vast majority of patients with pACC experienced distant versus local recurrence, with a distribution similar to those described in prior series of PDAC perhaps with an even higher proportion of isolated hepatic disease in the pACC cohort.^31,32^ The gap between RFS and OS suggests that a subset of these patients may have treatment responsive and relatively indolent biologic behavior with opportunities for salvage therapies potentially providing additional years of life despite recurrent disease. With careful selection in a multidisciplinary setting, surgery, liver-directed therapy, and other local therapies (i.e., radiation) may play a role in patients with oligometastatic disease, similar to that of the well differentiated neuroendocrine tumor counterparts.^33,34^ Median overall survival has not yet been met after a median follow up of 32 months (range: 10–62 months) from time of metastasectomy in the nine patients with resected oligometastatic disease in this study. Of note, sequencing was available for eight of the nine patients, of whom six had a mutation in the HRR pathway and two had TMB > 10, perhaps reflecting a possible positive predictive association of genetic mutations and favorable survival. Nevertheless, surgery for metastatic disease must be pursued with caution only in highly selected patients as prior studies have failed to report a statistical difference in survival after metastasectomy of pACC when compared with metastatic disease without surgery.^7^

Prior reports have suggested a higher prevalence of somatic or germline mutations in *BRCA2* and other HRR related genes in pACC tumors.^7,24,35,36^ Genetic analysis of a large pan-can cohort identified 36% of pACC to harbor germline pathogenic mutations in variants of homologous recombination related genes.^22^ Treatment with platinum-based chemotherapy and poly-ADP-ribose polymerase inhibitors (PARPi) have conveyed a survival advantage in PDAC patients with core HRR mutations.^18–21^ The APOLLO trial (NCT04858334) of adjuvant olaparib versus placebo in resected pancreas cancer, including pACC, with HRR mutations after completion of standard therapy is ongoing and may provide further clarity. Prior studies have suggested increased efficacy of platinum-based therapy in patients with metastatic pACC; the direct relationship of a survival benefit, platinum therapy, and an HRR mutation has yet to be clearly shown.^7,8,37^ Statistical significance of a survival advantage with adjuvant platinum therapy in this cohort of resected pACC with core HRR mutations was not achieved perhaps secondary to the overall small sample size or heterogeneity of chemotherapy. Of patients with a core HRR mutation, only one did not receive platinum-based therapy limiting the ability for additional comparisons within this small cohort. Furthermore, the retrospective nature of this study did not permit guidance of adjuvant therapy. Nevertheless, while resected tumor specimens were utilized for sequencing in this retrospective study, previous work has supported the utility of large-scale sequencing of tumors from tissue biopsies with a turnaround time that permits clinical interpretation and utilization.^23^ The 26% rate of core HRR mutations and 45% rate of any HRR-related mutation identified in this study offers intriguing insight for future consideration of systemic therapy, especially under the context of the response in other disease biologies with similar mutational status. With support from future clinical trials, an increase of routine genomic analysis in the preoperative setting may be useful in the guidance of targeted, effective neoadjuvant therapy.

The findings of this work must be viewed in the context of several limitations. Firstly, there are institutional practice pattern variations and changes across the time frame of this study. Next, only patients who proceeded to the operating suite and underwent curative-intent resection were included in analysis, excluding those with rapidly progressive or metastatic biology, which may be distinct from the resectable pACC cohort. Additionally, cases were abstracted retrospectively through pathology review leading to challenges in identifying a true denominator and accurately determining the incidence of this rare biology amongst all pancreatic cancers. Quantifying the effects of adjuvant therapy of any type are limited due to the retrospective biases of this study, the small sample size, and the heterogeneous chemotherapy treatments throughout the post operative period. Moreover, many patients opt for chemotherapy at local institutions where dosage and treatment details are difficult to obtain. The sequencing was not performed in a prospective fashion in all pACC tumors and patients; thus, treatment decisions were not routinely informed by sequencing data. Patients did not in all cases have routine germline testing to confirm the tumoral mutation findings. Furthermore, sequencing techniques were different across institutions and across the duration of the over 20-year period of this study opening the opportunity for differences in sequencing depth and sensitivity.

In conclusion, this study assists in the prognostication of patients with a rare diagnosis of pACC who are able to undergo resection, which yielded a favorable median OS of 73 months in this series. Mutations within the HRR pathway are frequently identified in pACC at rates perhaps 10× that of PDAC, providing opportunities for an informed selection of chemotherapy with platinum-based agents and PARPi. With this knowledge, the growing ease and decreasing cost of genetic sequencing, all patients with pACC, and their tumors should undergo sequencing to obtain information that could feasibly assist with treatment direction while also potentially identifying family members with HRR gene alterations of whom should undergo cancer screening. Future studies will further define and quantify the potential treatment advantage of targeted therapy in this select group of patients.

## Disclosure

Eileen O'Reilly: research Funding to institution provided by Genentech/Roche, BioNTech, AstraZeneca, Arcus, Elicio, Parker Institute, NIH/NCI, Digestive Care, Break Through Cancer, and Agenus; consulting/DSMB from Arcus, Ability Pharma, Alligator BioSciences, Agenus, BioNTech, Ipsen, Merck, Moma Therapeutics, Novartis, Syros, Leap Therapeutics, Astellas, BMS, Fibrogen, Revolution Medicines, Regeneron, and Merus; and conflicts of interest with Agios (spouse), Genentech-Roche (spouse), Eisai (spouse), and Servier (spouse). Lei Zheng receives grant support from Bristol-Myers Squibb, Merck, and Astrazeneca. Lei Zheng is a paid consultant/Advisory Board Member at Biosion, Alphamab, NovaRock, Ambrx, Akrevia/Xilio, QED, Amberstone, Tavotek Lab, Duo Oncology, ClinicalTrial Options, LLC, and Mingruizhiyao. Lei Zheng holds shares at Amberstone, Alphamab, Cellaration, and Mingruizhiyao. Alice Wei is a consultant for Histosonics and receives institutional clinical trial funding from Ipsen. Memorial Sloan Kettering has institutional financial interests in BioNTech, Epistem Prognostics, and Clarity Pharmaceuticals. This work was presented as an oral presentation at the 2024 AHPBA in Miami, Fl, USA.

## Supplementary Information

Below is the link to the electronic supplementary material.

Supplementary file1 (DOCX 76 KB)
