# Supplementary material for: Survival Outcomes and Genetic Characteristics of Resected Pancreatic Acinar Cell Carcinoma
Source: Ann Surg Oncol. 2024 Nov 22;32(3):1869–78. doi: 10.1245/s10434-024-16331-4 (PMC11811437; doi:10.1245/s10434-024-16331-4)
Supplement: Supplementary file 1 — Supplementary file1 (DOCX 76 KB) [file 10434_2024_16331_MOESM1_ESM.docx]

**Supplemental Figure 1.** Distribution of documented recurrence patterns after resection of pACC with Core HRR mutation

**
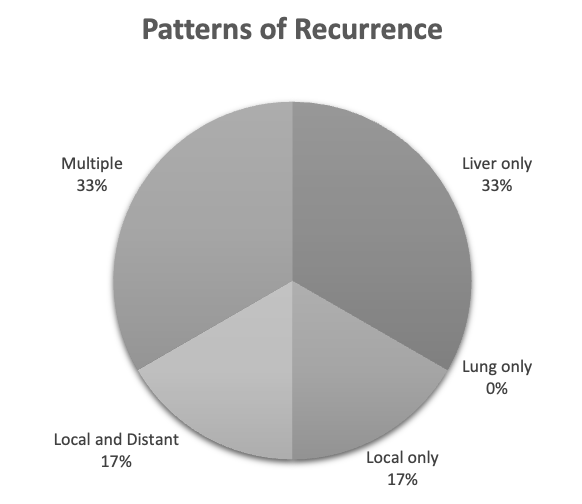
**
